# MR Imaging Features to Differentiate Retinoblastoma from Coats’ Disease and Persistent Fetal Vasculature

**DOI:** 10.3390/cancers12123592

**Published:** 2020-11-30

**Authors:** Robin W. Jansen, Christiaan M. de Bloeme, Hervé J. Brisse, Paolo Galluzzi, Liesbeth Cardoen, Sophia Göricke, Philippe Maeder, Nathalie Cassoux, Arnaud Gauthier, Sabrina Schlueter, Theodora Hadjistilianou, Francis L. Munier, Jonas A. Castelijns, Paul van der Valk, Annette C. Moll, Marcus C. de Jong, Pim de Graaf

**Affiliations:** 1 Department of Radiology and Nuclear Medicine, Amsterdam UMC, Vrije Universiteit Amsterdam, Cancer Center Amsterdam, 1081HV Amsterdam, The Netherlands; r.jansen1@amsterdamumc.nl (R.W.J.); c.debloeme@amsterdamumc.nl (C.M.d.B.); j.castelijns@amsterdamumc.nl (J.A.C.); mc.dejong@amsterdamumc.nl (M.C.d.J.); 2 Department of Radiology, Institut Curie, Paris University, 75005 Paris, France; herve.brisse@curie.fr (H.J.B.); liesbeth.cardoen@curie.fr (L.C.); 3 Department of Neuroimaging and Neurointervention, Siena University Hospital, 53100 Siena, Italy; galluzzip@libero.it; 4 Department of Diagnostic and Interventional Radiology and Neuroradiology, University Hospital Essen, 45147 Essen, Germany; sophia.goericke@uk-essen.de; 5 Department of Radiology, Centre Hospitalier Universitaire Vaudois (CHUV), University of Lausanne, 1011 Lausanne, Switzerland; philippe.maeder@chuv.ch; 6 Department of Ocular Oncology, Institut Curie, Paris University, 75005 Paris, France; nathalie.cassoux@curie.fr; 7 Department of Pathology, Institut Curie, Paris University, 75005 Paris, France; arnaud.gauthier@curie.fr; 8 Department of Ophthalmology, University Hospital Essen, 45147 Essen, Germany; sabrina.schlueter@uk-essen.de; 9 Department of Ophthalmology, Siena University Hospital, 531000 Siena, Italy; dorisocularoncology@libero.it; 10 Department of Ophthalmology, Centre Hospitalier Universitaire Vaudois (CHUV), University of Lausanne, 1011 Lausanne, Switzerland; francis.munier@fa2.ch; 11 Hôpital Ophtalmique Jules-Gonin, 1004 Lausanne, Switzerland; 12 Department of Pathology, Amsterdam UMC, Cancer Center Amsterdam, Vrije Universiteit Amsterdam, 1081HV Amsterdam, The Netherlands; p.vandervalk@amsterdamumc.nl; 13 Department of Ophthalmology, Amsterdam UMC, Cancer Center Amsterdam, Vrije Universiteit Amsterdam, 1081HV Amsterdam, The Netherlands; a.moll@amsterdamumc.nl

**Keywords:** retinoblastoma, pseudoretinoblastoma, Coats’ disease, persistent fetal vasculature, MRI

## Abstract

**Simple Summary:**

Retinoblastoma is a rare cancer that develops in the retina of children. Accurate differentiation between retinoblastoma and conditions that show similarities with retinoblastoma (pseudoretinoblastoma or retinoblastoma mimickers) is vital for guiding treatment. The most common pseudoretinoblastoma conditions are Coats’ disease and persistent fetal vasculature (PFV). In this study, we aimed to improve pretreatment differentiation between these diseases on MR imaging. We compared pre-treatment MR images of retinoblastoma, Coats’ disease and PFV on 20 predefined MR imaging features. An assessment strategy was proposed incorporating MR imaging features that best differentiate retinoblastoma from pseudoretinoblastoma, including three newly identified MR imaging features.

**Abstract:**

Retinoblastoma mimickers, or pseudoretinoblastoma, are conditions that show similarities with the pediatric cancer retinoblastoma. However, false-positive retinoblastoma diagnosis can cause mistreatment, while false-negative diagnosis can cause life-threatening treatment delay. The purpose of this study is to identify the MR imaging features that best differentiate between retinoblastoma and the most common pseudoretinoblastoma diagnoses: Coats’ disease and persistent fetal vasculature (PFV). Here, six expert radiologists performed retrospective assessments (blinded for diagnosis) of MR images of patients with a final diagnosis based on histopathology or clinical follow-up. Associations between 20 predefined imaging features and diagnosis were assessed with exact tests corrected for multiple hypothesis testing. Sixty-six patients were included, of which 33 (50%) were retinoblastoma and 33 (50%) pseudoretinoblastoma patients. A larger eye size, vitreous seeding, and sharp-V-shaped retinal detachment were almost exclusively found in retinoblastoma (*p* < 0.001–0.022, specificity 93–97%). Features that were almost exclusively found in pseudoretinoblastoma included smaller eye size, ciliary/lens deformations, optic nerve atrophy, a central stalk between optic disc and lens, Y-shaped retinal detachment, and absence of calcifications (*p* < 0.001–0.022, specificity 91–100%). Additionally, three newly identified imaging features were exclusively present in pseudoretinoblastoma: intraretinal macrocysts (*p* < 0.001, 38% [9/24] in Coats’ disease and 20% [2/10] in PFV), contrast enhancement outside the solid lesion (*p* < 0.001, 30% [7/23] in Coats’ disease and 57% [4/7] in PFV), and enhancing subfoveal nodules (38% [9/24] in Coats’ disease). An assessment strategy was proposed for MR imaging differentiation between retinoblastoma and pseudoretinoblastoma, including three newly identified differentiating MR imaging features.

## 1. Introduction

Retinoblastoma, like other intra-ocular tumors, are diagnosed and treated without upfront histopathologic confirmation. Clinicians are largely dependent on extensive ophthalmologic examination (including fundoscopy under general anesthesia, optical coherence tomography, fluorescein angiography) and both US and MR imaging. However, some benign conditions show many similarities with retinoblastoma in their clinical and imaging presentation: ‘retinoblastoma mimickers’, ‘retinoblastoma simulating lesions’, or ‘pseudoretinoblastoma’. Of patients referred with suspicion of retinoblastoma, 16–22% have simulating lesions [1,2]. Of these, Coats’ disease (40%) and persistent fetal vasculature (PFV) (26%) are the two most common conditions mimicking retinoblastoma [2], often presenting in young children with leukocoria as the first symptom.

Coats’ disease is a pediatric unilateral vascular retinal disorder, associated with retinal telangiectasia, sub- and intra-retinal exudates and retinal detachment. Coats’ disease has five progressive stages, of which especially advanced stage (3–5) Coats’ disease can mimic retinoblastoma. PFV is a uni- or bi-lateral congenital disorder characterized by incomplete involution of the embryological hyaloid vasculature resulting in a fibrovascular stalk connecting the retrolental membrane to the retina. The stalk may pull on surrounding structures causing retinal folds, tractional retinal detachment, retinal or vitreous hemorrhages, retinal dysplasia, or hypoplasia of the optic nerve and macula. Retinal dysplasia can be present within the spectrum of findings in PFV but can also present itself as a separate diagnostic entity in 3% of pseudoretinoblastoma [2,3,4].

Although differentiation between retinoblastoma and pseudoretinoblastoma can be challenging [3,4,5,6,7,8,9,10,11,12], it is vital for initiating adequate treatment. A missed retinoblastoma diagnosis can cause life-threatening treatment delay, while a false-positive retinoblastoma diagnosis can induce mistreatment including chemotherapy [7,8,9,10,11,12]. Additionally, correct classification can reduce unnecessary enucleations, as 5–12% of suspected retinoblastoma appear to be pseudoretinoblastoma upon histopathologic assessment [13,14]. However, it is important to mention that the misdiagnosis rate has been decreasing [14] and that enucleation is still a valid option in both advanced retinoblastoma and pseudoretinoblastoma, for example in advanced Coats’ disease.

MR imaging is generally requested for patients with uncertain diagnosis after extensive clinical assessment including ultrasound examination. MR imaging also plays a role in the work-up of patients with retinoblastoma for depicting disease extent and screening the central nervous system for trilateral retinoblastoma or metastasis. MR imaging features of retinoblastoma and pseudoretinoblastoma have been described. The smaller size of the affected eye (compared with the contralateral) on MR imaging ranges from PFV eyes (smallest, regularly microphtalmia with >2SD below mean size for age), to Coats’ disease eyes (smaller than retinoblastoma eyes, but no microphtalmia), to retinoblastoma (mean size slightly smaller than healthy eyes) [15,16]. Hyperintensity (on T1, T2 and FLAIR images) of subretinal exudate has been described in advanced Coats’ disease [17,18,19]. MR imaging of PFV shows intra-ocular hemorrhage, lens deformations, vitreous membranes from the optic nerve (residual fetal hyaloid canal), and retinal detachment in a T-/funnel-shape or Y-shape (sometimes called ‘Martini-Glass-Sign’) [20,21]. However, imaging characteristics of pseudoretinoblastoma and retinoblastoma have not been directly compared, and the diagnostic accuracy of imaging features for disease differentiation is uncertain.

The hypothesis of this study was that MR imaging features can aid discrimination between retinoblastoma and common pseudoretinoblastoma. The purpose of this study was to identify MR imaging features that best differentiate between retinoblastoma and the most common pseudoretinoblastoma diagnoses: Coats’ disease and persistent fetal vasculature (PFV).

## 2. Results

### 2.1. Patient Populations and MR Images

Sixty-eight eyes from 66 patients were included, of which 33 (50%) were retinoblastoma patients and 33 (50%) pseudoretinoblastoma patients. Patients were included from four retinoblastoma referral centers: 37 from Amsterdam (the Netherlands), 13 from Paris (France), 11 from Siena (Italy) and 5 from Essen (Germany), characteristics summarized in Table 1. Scan dates ranged from 1997 to 2019 (median 2014); mean MR image quality was 2.7/5. Interobserver agreement for 6 reviewers varied between features with Kappa values ranging from 0.15 to 0.71 (Table 2 and Table S1).

### 2.2. Imaging Features’ Associations with Diagnosis

Diagnosis was correct in 99% (67/68) of consensus scores and 77% (314/408) of individual assessments (range 47–97%). Fifteen out of 20 (75%) imaging features showed significant association with diagnosis, of which 11/20 (55%) of features remained significant after adjusting to multiple hypothesis testing (Table 2 and Table S1).

### 2.3. Imaging Features Predicting Retinoblastoma or Pseudoretinoblastoma

Of the imaging features that were significantly associated with diagnosis, sensitivity and specificity are available in Table S2. Features with a sensitivity, specificity or accuracy over 90% are listed in Table 3.

A larger eye size, narrow v-shaped detachment, and lesion islands in the vitreous (seeding) were almost exclusively found in retinoblastoma and therefore showed high specificity (93–97%), but sensitivity was low (9–30%).

Features that were almost exclusively present in (i.e., highly specific for) pseudoretinoblastoma were a smaller eye size, Y-shaped retinal detachment, absence of calcifications, intraretinal macrocysts, no/minimal contrast enhancement within solid component, contrast enhancement outside the solid lesion (mismatch), ciliary body deformation, lens deformations, optic nerve atrophy and central stalk between optic disk and lens (specificity 91–100%). The sensitivity of these features ranged from 22 to 62%, and accuracy ranged from 62% to 83%.

Within these features guiding towards pseudoretinoblastoma, there were features that were almost exclusively present in one specific pseudoretinoblastoma diagnosis. Optic nerve atrophy was exclusively found in PFV (specificity of 100%), and a central stalk (optic disc to lens) was almost exclusively found in PFV (specificity 98%). Intraretinal macrocysts were most frequently found in Coats’s disease in 38% (9/24) of eyes, versus 20% (2/10) of PFV and 0% (0/34) of retinoblastoma eyes, with a specificity of 95%. Subretinal hyperintensity (compared with vitreous) was not significantly associated with Coat’s disease. This applied to both T1-weighted images (Coats’ 96% [22/23] vs retinoblastoma 94% [29/31] vs PFV 100% [9/9]) and T2-weighted images (Coats’ 4% [1/23] vs retinoblastoma 0% [0/31] vs PFV 0% [0/9]). 

### 2.4. Three Newly Identified MR Imaging Features in Coats’ Disease and PFV/Retinal Dysplasia

**Figure 1:**
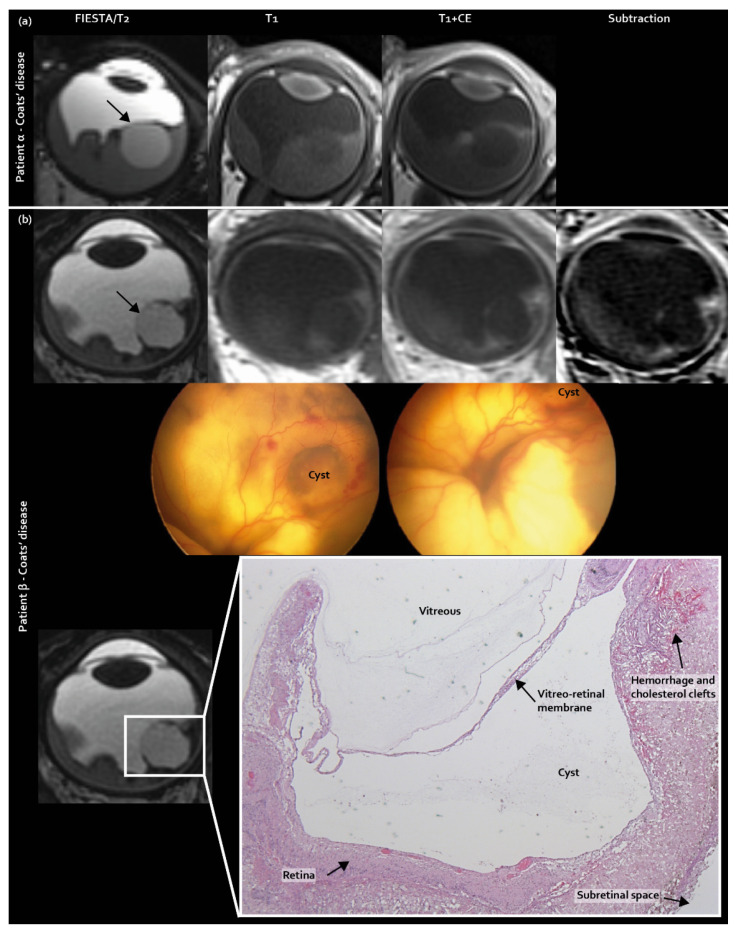
Intraretinal macrocysts as a newly identified MR imaging feature exclusively found in pseudoretinoblastoma (Coats’ disease and PFV/retinal dysplasia): (**a**) MR imaging of an intraretinal macrocyst in Coats’s disease patient α; (**b**) MR imaging of an intraretinal macrocyst with corresponding Retcam fundus camera images and histopathology of Coats’ disease patient β.

Three new features were identified as guiding towards pseudoretinoblastoma diagnosis: intraretinal macrocysts, contrast enhancement outside the solid lesion, and subfoveal enhancing nodules. Intraretinal macrocysts, incorporated in the scoring list, were found in 38% (9/24) of Coats’ disease eyes, compared with 20% (2/10) of PFV/retinal dysplasia eyes and 0% (0/34) of retinoblastoma eyes (corrected *p* = 0.005, Figure 1).

**Figure 2:**
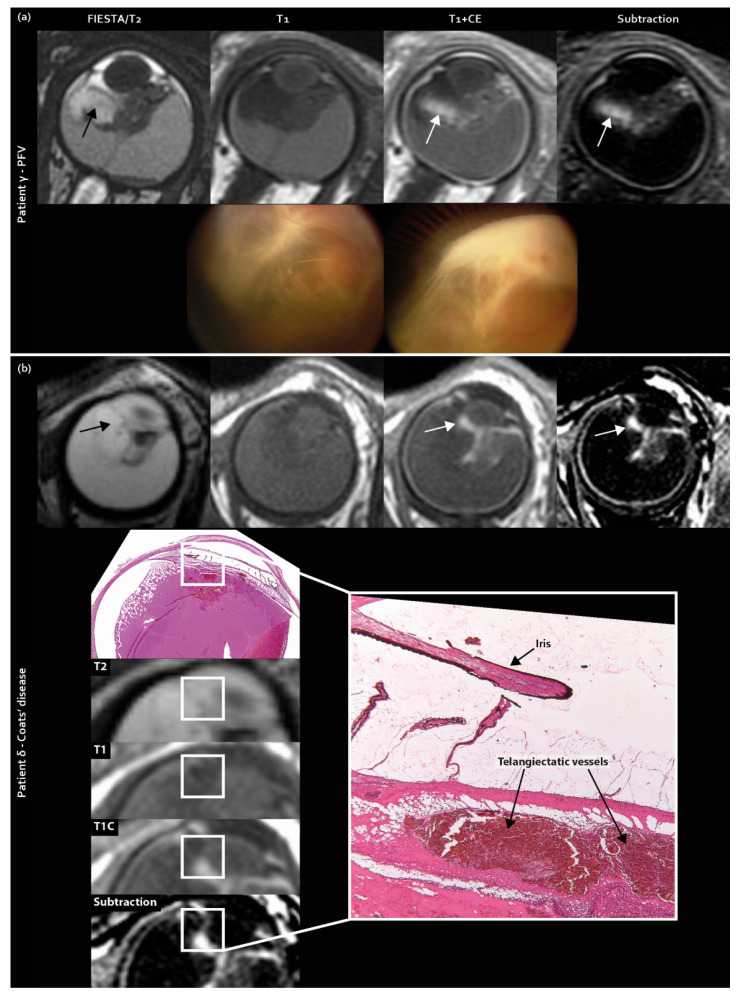
Contrast enhancement outside the solid lesion (enhancement mismatch) as a newly identified MR imaging feature exclusively found in pseudoretinoblastoma (Coats’ disease and PFV/retinal dysplasia): (**a**) MR imaging of enhancement mismatch and corresponding Retcam fundus camera images showing elongated ciliary body processes in PFV patient γ; (**b**) MR imaging of enhancement mismatch with corresponding histopathology in Coats’ disease patient δ.

Contrast enhancement outside the solid mass lesion (T2-hypointense) was also included in the scoring list as a new imaging feature and was exclusively found in pseudoretinoblastoma (Coats’ disease 30% [7/23], PFV 57% [4/7] retinoblastoma 0% [0/34], *p* < 0.001 (Figure 2)).

**Figure 3:**
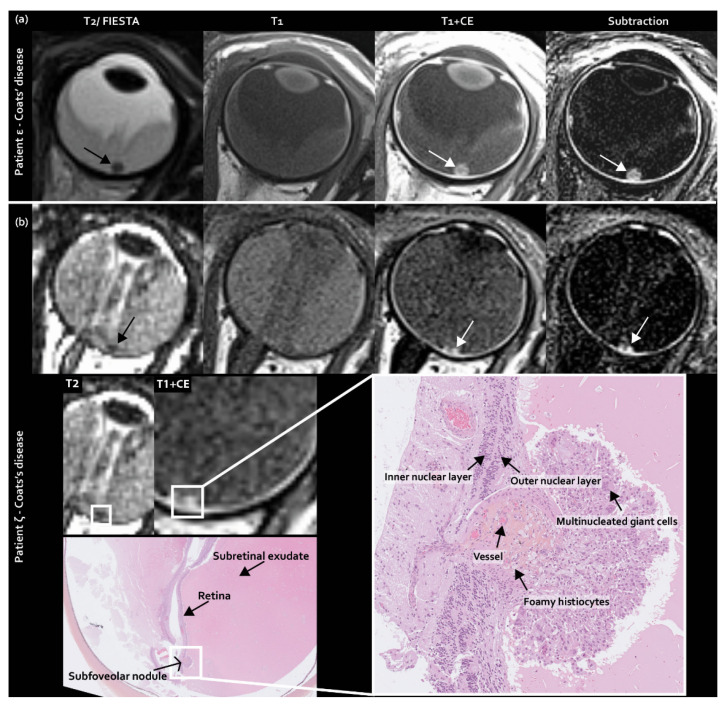
Subfoveal enhancing nodules as a newly identified MR imaging feature exclusively found in Coats’ disease: (**a**) MR imaging of (enhancing) subfoveal nodules in Coats’ disease patient ε; (**b**) MR imaging of (enhancing) subfoveal nodules with corresponding histopathology of Coats’ disease patient ζ.

Next to typically non-enhancing tumor seeding in the subretinal space in retinoblastoma, as incorporated in the predefined imaging features list, subretinal enhancing nodules were encountered at the fovea. These nodules appeared to be present only in Coats’ disease in 33% (8/24) of eyes upon post-hoc analysis (Figure 3) and were congruent with subfoveal nodules, as described in ophthalmologic Coats’ disease literature [22].

### 2.5. Assessment Strategy for Differentiating Retinoblastoma, Coats’ Disease and PFV/Retinal Dysplasia

As a proposed assessment strategy, imaging features were selected based on a statistically significant associations with a specific diagnosis combined with high specificity (>90%). The feature ‘enhancing subfoveal nodules’, identified in post-hoc analysis, was added.

**Figure 4:**
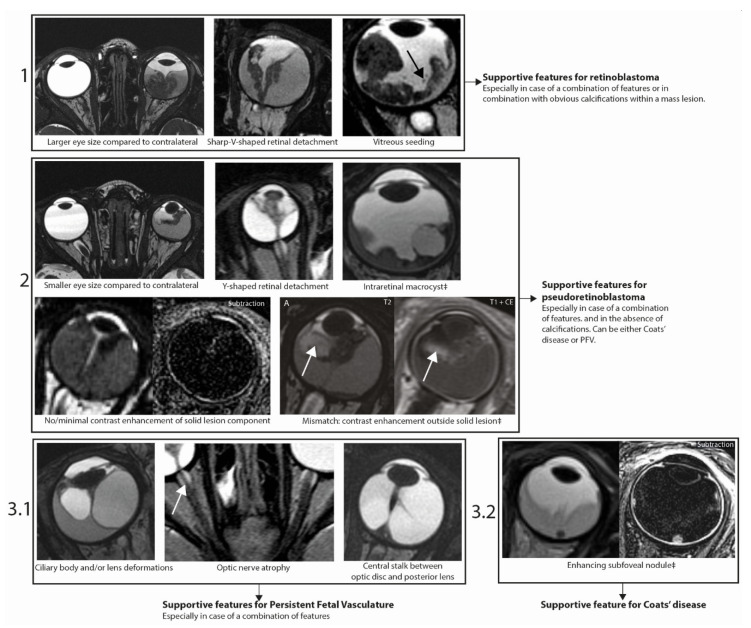
Assessment strategy for differentiation of retinoblastoma, Coats’ disease and PFV/retinal dysplasia on MR Imaging. The strategy includes three newly identified imaging features: intraretinal macrocysts, contrast enhancement outside the solid lesion and enhancing subfoveal nodules (new features indicated by ‡). All features were statistically significantly associated with diagnosis (corrected for multiple testing) and showed high specificity (≥90%). All example images are CISS/FIESTA images unless otherwise specified in the top right corner.

Firstly, a larger eye size compared with contralateral, a sharp-V-shaped retinal detachment, and lesion islands in vitreous (seeding) are supportive features for retinoblastoma (Figure 4, step 1). Secondly, a (combination of a) significantly smaller eye size compared with contralateral, Y-shaped retinal detachment, absence of calcifications, intraretinal macrocysts, absence of contrast enhancement within the solid lesion and contrast enhancement outside the solid lesion strongly support pseudoretinoblastoma—either Coats’ disease or PFV/retinal dysplasia (Figure 4, step 2). Thirdly, ciliary body and/or lens deformations, optic nerve atrophy and a central stalk between the optic disc and the lens specifically support diagnosis of PFV/retinal dysplasia (Figure 4 step 3.1), while subretinal enhancing lesions support diagnosis of Coats’ disease (Figure 4, step 3.2). Features should be assessed in combination. Decisions on diagnosis should not be made based on individual traits, especially features with specificity <97% such as smaller eye size indicating pseudoretinoblastoma (specificity 91%) and larger eye size indicating retinoblastoma (specificity 93% and 96%, respectively).

## 3. Discussion

Retinoblastoma is a clinical diagnosis that is made through ophthalmologic examination, including fundoscopy and ultrasound examination. For differentiation between retinoblastoma and pseudoretinoblastoma, ultrasonography can aid through detection of calcifications, generally present (95%) in retinoblastoma, occasionally present in advanced Coats’ disease and not known to be present in PFV [23]. The uncommon diffuse infiltrative retinoblastoma subtype commonly contains no calcifications and may therefore mimic Coats’ disease or PFV [24]. MR Imaging is recommended for (suspected) retinoblastoma to assess disease extent, estimate metastatic risk and to screen the CNS for metastasis or *RB1* mutation associated with intracranial neuroectodermal tumors (trilateral retinoblastoma). Furthermore, MR imaging can be used to narrow down differential diagnosis in patients with leukocoria either to confirm retinoblastoma or to support the ophthalmologist in making the correct diagnosis. For (suspected) retinoblastoma, a diagnostic MR imaging protocol including high-resolution contrast enhanced images is recommended [25]. Differentiation between retinoblastoma, Coats’ disease and PFV/retinal dysplasia can in some cases be notoriously hard due to a similar appearance in clinical, fundoscopic, and MR imaging presentation. Although this issue and possible distinguishing imaging features have been discussed before [17,26,27], previous reports are limited to descriptive reviews and expert opinions with no direct comparison of cases in a standardized research setting. In this study, fourteen differentiating imaging features were identified through the standardized scoring of MR images. Differentiating features, including three newly-identified features, were incorporated in a proposed MR imaging assessment strategy with the goal of diminishing the amount of cases with diagnostic uncertainty (Figure 4). Importantly, subgroups of included pathologies (i.e., retinoblastoma with an endophytic growth pattern and severe microphtalmic eyes in PFV) were excluded, since they show distinct phenotypic differentiating features and are less likely to be misdiagnosed during clinical and imaging workup. Additionally, only cases in which MR imaging was obtained were included, resulting in a selection of cases with diagnostic uncertainty based on clinical assessment alone.

Three new differentiating features were identified in this study. Firstly, intraretinal macrocysts were frequently found in pseudoretinoblastoma, predominantly in Coats’ disease. In Coats’ disease, cysts are caused by the exudation of lipids from abnormal vessels and have been reported in 11% of Coats’ disease cases in ophthalmological and pathology reports [28]. Cysts are related to prolonged retinal detachment and probably progress from more subtle intraretinal exudation (they have a prevalence of 35% in stage 2B Coats disease), towards intraretinal macrocysts that can be depicted by MR imaging. Cysts that are located intralesionally can be encountered in other retinal masses, including medulloepithelioma and cavitary retinoblastoma. The latter is an uncommon presentation of a well-differentiated retinoblastoma characterized by an endophytic growth pattern and cysts located intralesionally [29].

Secondly, contrast enhancement outside the solid area of the lesion (enhancement mismatch) was exclusively found in pseudoretinoblastoma, which can be explained by the increased leakage of contrast material from abnormal retinal vessels. In retinoblastoma, contrast enhancement is restricted to the retinal tumors, whereas the unaffected retina does not enhance. However, in pseudoretinoblastoma, vascular abnormalities are present throughout the retina (i.e., retinal telangiectasia in Coats’ disease) with a more wide-spread pattern of contrast leakage not necessarily confined to the retinal mass lesion [28]. The inter-rater agreement was poor for this MR imaging feature (enhancement outside the solid lesion), but post-hoc analysis showed that of the times any individual reader scored the presence of this feature, 42 out of the 44 times (96%) it involved a pseudoretinoblastoma diagnosis. The feature is not often detected in consensus by all readers, possibly due to its novelty (and hence showing a low agreement), but it is hardly ever encountered in retinoblastoma. This feature may show better agreement and discrimination performance when readers gain experience assessing it.

Thirdly, enhancing subfoveal nodules were newly identified on MR imaging in Coats’ disease, although a prevalence of 53% is reported in ophthalmologic and histopathologic assessments [22]. Histopathologically, subfoveal nodules contain protein- and lipid-rich material, and they progress over time into macular fibrosis, associated with worse visual outcomes [22]. The subfoveal nodules in this study showed various amounts of enhancement, which was possibly correlated to the degree of fibrosis. Potentially, subfoveal nodules could mimic subretinal tumor seeding in retinoblastoma. However, the key-features to differentiate these two entities are that tumor seeding does not enhance and is not preferentially located at the fovea. The imaging finding of a subfoveal enhancing nodule may therefore provide an important sign in favor of Coats’ disease. In the differential diagnosis, an enhancing granuloma in toxocara endophthalmitis could be considered in the correct clinical context [23].

Some features were previously attributed to one diagnosis, but their value in narrowing down the differential diagnosis was not confirmed in this study. Hyperintensity of subretinal fluid, which is commonly linked to Coats disease in the literature, was present in the majority of included patients (all diagnoses) and therefore does not provide adequate discriminative potential. Additionally, although bilaterality can be generally regarded as a sign of retinoblastoma, the finding should be interpreted with care because bilateral pseudoretinoblastoma cases do occasionally exist both in the literature and the current analysis [30]. Finally, the presence of calcifications is regarded as pathognomonic for retinoblastoma, and spots of signal voids were detected in all retinoblastoma cases. For ruling out an alternative diagnosis, however, calcifications can be misleading because results indicate that in pseudoretinoblastoma lesions resembling calcifications are frequently present. Possibly, this is due to a focal hemorrhage causing signal intensity voids similar to calcifications [31]. Furthermore, the absence of calcifications as solitary findings does not rule out retinoblastoma since occasionally retinoblastoma presents itself at a relatively older age, with diffuse infiltrative tumors showing minimal tumor mass without calcifications [24,32].

This study yielded limitations, which can in part be attributed to the rarity of the diseases and subsequent retrospective design. In order to collect a reasonable number of patients, a wide range of scan dates and scan qualities were included, possibly diminishing power for features requiring high spatial resolution. However, the study included cases of many different scanner types and scanning protocols, bring about generalizable results for different settings and institutions. The readers’ agreement varied extensively and was low for imaging features that require high spatial resolution (e.g., ciliary body deformations) and for the newly described imaging features. This identifies an important issue for the application of the outcomes of this study in clinical practice, where imaging is mostly assessed on an individual basis. For the novel MR imaging features, readers may gain experience over time, resulting in an improved inter-reader agreement. The example images and assessment strategy might assist in familiarizing readers with the imaging features, potentially increasing inter-reader agreement for future readers. An additional limitation of this multicenter study is that results were not validated in an independent cohort or by an independent group of radiologists. Although the current setting enabled identification of differentiating MR imaging features, a prospective study would be better suited for assessing their accuracy. Finally, ultrasonography features could not be included in this retrospective imaging analysis. Ultrasonography is also a powerful imaging tool because of its high spatial resolution, high sensitivity to depicting calcifications typical for retinoblastoma, and its ability to assess vascularized tissues or persistent hyaloid vasculature by Doppler analysis [19,23,25]. Furthermore, ultrasonography is a cost-effective, non-invasive imaging method that is widespread and available in different settings without the need for general anesthesia. A close collaboration with the ophthalmologist, taking into account clinical features, fundoscopy and ophthalmic imaging modality features (i.e., ultrasonography, optical coherence tomography, fluorescence angiography) is vital for correct classification of these lesions. Several items that are usually assessed clinically were not considered here but are of vital importance to take into account, for example the laterality of disease.

Although the prevalence of certain individual imaging features was low (reflected by a low sensitivity) for both retinoblastoma predictors and pseudoretinoblastoma predictors, high specificity does indicate the usefulness of these features in clinical practice when present. The proposed assessment strategy may aid in preventing both false-negative retinoblastoma diagnosis causing treatment-delay and false-positive diagnosis causing mistreatment including chemotherapy. 

## 4. Materials and Methods

### 4.1. Patient Samples

This multicenter retrospective study was approved by the Institutional Review Board with a waiver of informed consent (no 2019.406), carried out following the rules of the Declaration of Helsinki of 1975 and reported in accordance with the Standards for Reporting of Diagnostic Accuracy Studies’ (STARD) 2015 guidelines [33]. Chart review was used to select patients with retinoblastoma, Coats’ disease or PFV/retinal dysplasia. For collecting MR images of these rare diseases, a long (25 years) period was adopted for retrospective selection of patients in European retinoblastoma referral centers using convenience sampling. By including patients with MR imaging present, only patients with uncertain diagnosis based on clinical assessment including ultrasonography were selected. In order to identify MR imaging features with added value for differentiation between the diseases, we aimed to select patients with (most) diagnostic uncertainty with current MR imaging assessment methods. To this extent, some subtypes of the diseases were included or excluded. For retinoblastoma, only exofytic retinoblastoma or diffuse-growing retinoblastoma were included because these growth types present retinal detachment and retinal thickening similar to pseudoretinoblastoma. Exofytic or diffuse growth patterns can be present at all retinoblastoma stages. For Coats’ disease, advanced stage disease (stage 3–5) patients were included because they can mimic retinoblastoma with similar retinal detachments. For PFV, eyes with severe microphtalmia were excluded because eyes with this finding are less likely to be difficult to distinguish from retinoblastoma eyes. Phtisis bulbi was excluded because no intra-ocular features could be assessed. For this study, PFV and retinal dysplasia were combined because they show overlapping clinical and imaging signs [3,4]. Dysplastic retinal tissue is a common finding in patients with PFV and can also be present as a separate entity. However, dysplastic retinal tissues as a separate entity or within the spectrum of PFV abnormalities are not known to present themselves with different MR imaging features, which substantiated the choice to jointly analyze these diseases for this imaging study. Differentiation between pseudoretinoblastoma and retinoblastoma can be very challenging for the described disease subtypes, mostly due to similar retinal detachment and retinal thickening patterns [6,11,34].

Therefore, the inclusion criteria consisted of: (i) diagnosis of advanced Coats’ disease or PFV/retinal dysplasia without severe microphtalmia or retinoblastoma with exofytic or diffuse growth type; (ii) diagnosis was based on (a combination of) histopathology, clinical findings during follow-up, fundoscopic presentation or fluorescence-angiography; and (iii) pre-treatment (baseline) MR imaging was available with at least T2-weighted and T1-weighted images (before and after intravenous gadolinium administration). The rarity of the diseases necessitated inclusion of a wide range of different MR images from various manufacturers with ranging quality. Patients were excluded if the MR images dated from before 1995 or the quality was scored below two on a Likert scale of 1–5. Seven of the 66 patients had been previously reported. Six patients were included in articles that dealt with genomics or radiogenomics of retinoblastoma [34,35], and one patient was described in a case report of retinal dysplasia mimicking an intraocular tumor [3], whereas in this study we report on differentiating imaging features between retinoblastoma and pseudoretinoblastoma.

### 4.2. MR Imaging Assessment

The validated “Retinoblastoma Imaging Atlas” was used as a basis for scoring the images [34], supplemented with imaging features suitable for Coats’ Disease and PFV/retinal dysplasia. Prior selection of these additional features was based on review of the literature [6,15,17,18,19,20,21,23] or expert radiologists’ experience (a full predefined imaging feature list including illustrations is available in Figure S1). Although laterality is an important factor for differentiation between diseases in clinical practice, it was not considered in this MR imaging feature list because it can be reliably assessed through clinical examination and ultrasonography. Six readers (H.J.B., P.G., S.G., P.M., M.C.d.J., P.d.G.) from the European Retinoblastoma Imaging Collaboration (ERIC) with 7–22 years of individual experience in the interpretation of ocular MR imaging independently scored the MR images, blinded for diagnosis and clinical details. Additionally, scan quality was scored on a Likert scale of 1–5. Readers were offered the option ‘unassessable’ for each feature, which was scored in the case of inadequate imaging quality for an individual feature or if features could not be assessed correctly (e.g., unassessable ‘shape of retinal detachment’ in absence of retinal detachment). Readers scored MR imaging features, blinded for diagnosis and for each other’s scores, and discrepancies were resolved by consensus.

### 4.3. Statistical Analysis

Interobserver agreement for MR imaging features was assessed by calculating Fleiss’ Kappa. The Fisher-Freeman-Halton exact test (two-sided) was used for comparing MR features and diagnosis. *p*-values were Bonferonni-corrected for multi-hypothesis testing. Sensitivity and specificity (including 95% confidence intervals (95%CI)) were calculated to assess the accuracy of MR imaging features used to differentiate between retinoblastoma and mimickers.

## 5. Conclusions

This study identified MR imaging features that help differentiate retinoblastoma from Coats’ disease and PFV. An MR imaging assessment strategy was proposed, including three newly identified imaging features exclusively found in pseudoretinoblastoma: intraretinal macrocysts, contrast enhancement outside the solid lesion, and (enhancing) subfoveal nodules.

## Figures and Tables

**Table 1:** Patient demographics.

	Retinoblastoma	Coats’ Disease	PFV/Retinal Dysplasia
Patients *n* (%)	33 (50%)	24 (36%)	9 (14%) (of which *n* = 2 isolated retinal dysplasia)
Assessed eyes *n* (%)	34 (50%)	24 (35%)	10 (15%)
Laterality OD/OS/ODS	15/17/1	10/14/0	3/5/1
Stage ^1^	B 1/27 (4%) C: 4/27 (15%) D: 7/27 (26%) E: 15/27 (56%)	Advanced stage (3–5): 24/24 (100%)	-
Female/male (%)	15/18 (45/55%)	3/21 (13/88%)	2/7 (22/78%)
Mean age at MRI scan in months (standard deviation)	34 (27)	38 (30)	16 (24)
Mean follow-up duration in months ^2^ (standard deviation)	59 (41)	16 (27)	13 (16)
Mean scan year (range)	2013 (1997–2019)	2013 (2009–2019)	2014 (1999–2019)

^1^ Staging was based on International Classification of Retinoblastoma (ICRB) for retinoblastoma and Shields’ classification of Coats’ disease; for staging of persistent fetal vasculature (PFV)/retinal dysplasia, no specific staging system was known. Retinoblastoma information on staging was missing in 20% (7/34) of eyes. ^2^ Data were missing in 8% (5/66) of patients.

**Table 2:** MR Imaging features significantly associated with diagnosis (retinoblastoma, Coats’ disease, PFV/retinal dysplasia).

Imaging Feature	Retinoblastoma ^1^	Coats’ Disease ^1^	PFV/ Retinal Dysplasia ^1^	Pseudoretinoblastoma (Coats’ Disease and PFV)	K ^2^	*p* ^3^
**Eye size ^4^**					0.37	<0.001
Smaller	9% (3/33)	67% (16/24)	80% (8/10)	71% (24/34)		
Equal	82% (27/33)	33% (8/24)	10% (1/10)	26% (9/34)		
Larger	9% (3/33)	0% (0/24)	10% (1/10)	3% (1/34)		
**Ciliary deformations**	0% (0/34)	13% (3/24)	78% (7/9)	30% (10/33)	0.39	<0.001
**Lens deformations**	3% (1/34)	17% (4/24)	90% (9/10)	38% (13/34)	0.53	<0.001
**Optic nerve atrophy**	0% (0/34)	0% (0/24)	70% (7/10)	21% (7/34)	0.49	<0.001
**Central stalk ^5^**	0% (0/34)	4% (1/23)	70% (7/10)	24% (8/33)	0.43	<0.001
**Shape of retinal detachment ^6^**					0.40	0.007
T	22% (7/32)	25% (5/20)	44% (4/9)	31% (9/29)		
Y	0% (0/32)	20% (4/20)	44% (4/9)	28% (8/29)		
Sharp-V	25% (8/32	5% (1/20)	11% (1/9)	7% (2/29)		
Wide-V	53% (17/32)	50% (10/20)	0% (0/9)	34% (10/29)		
**Intraretinal macrocyst**	0% (0/34)	38% (9/24)	20% (2/10)	32% (11/34)	0.55	0.005
**Absence of calcifications**	0% (0/34)	67% (14/21)	50% (4/8)	62% (18/29)	0.35	<0.001
**Lesion islands/seeding**					0.44	<0.001
Vitreous	30% (10/33)	4% (1/23)	0% (0/10)	3% (1/33)		
Subretinal	64% (21/33)	30% (7/23)	10% (1/10)	24% (8/33)		
**No/minimal enhancement in solid lesion**	3% (1/34)	31% (7/22)	57% (4/7)	38% (11/29)	0.35	0.008
**Enhancement outside solid lesion (mismatch)**	0% (0/34)	30% (7/23)	57% (4/7)	37% (11/30)	0.15	<0.001

^1^ If eyes do not add up to *n* = 34 retinoblastoma, *n* = 24 Coats’ disease, and *n* = 10 PFV/retinal dysplasia, cases were in consensus scored as unassessable; ^2^ Fleiss’ Kappa for interreader agreement; ^3^ Bonferroni-corrected *p*-value derived from two-sided Fisher Exact or Fisher-Freeman-Halton tests for association with diagnosis. Only significant features are shown, but other analyses are available in Table S1. ^4^ Eye size of (most) affected eye was compared with contralateral; ^5^ central stalk from optic disc to lens; ^6^ see Figure S1.

**Table 3:** Sensitivity, specificity and accuracy, including 95% confidence intervals (CI), for differentiating retinoblastoma from pseudoretinoblastoma (Coats’ disease or PFV/retinal dysplasia) or one pseudoretinoblastoma diagnosis from the other diagnoses (e.g., PFV vs retinoblastoma and Coats’ disease).

Features Favoring	Imaging Feature ^1^		Sensitivity (95%CI)	Specificity (95%CI)	Accuracy (95%CI)
Retinoblastoma	Larger eye size ^2^		9% (2–25%)	**97% (85–100%)**	54% (41–66%)
	Narrow V-shape		25% (11–43%)	**93% (77–99%)**	57% (44–70%)
	Lesion islands/vitreous		30% (16–49%)	**96% (78–100%)**	58% (44–71%)
Pseudoretinoblastoma	Smaller eye size ^1^		71% (53–85%)	**91% (76–98%)**	81% (69–89%)
	Y-shaped retinal detachment		28% (13–47%)	**100% (89–100%)**	65% (52–77%)
	Absence of calcifications		62% (42–79%)	**100% (90–100%)**	83% (81–91%)
	Intraretinal macrocysts		32% (17–51%)	**100% (90–100%)**	66% (54–77%)
	No/minimal enhancement in solid lesion		38% (21–57%)	**97% (85–100%)**	70% (57–81%)
	Enhancement outside solid lesion (mismatch)		37% (20–56%)	**100% (90–100%)**	70% (58–81%)
	Ciliary body deformations		30% (65–93%)	**100% (89–100%)**	66% (53–77%)
	Lens deformations		38% (22–56%)	**97% (84–100%)**	68% (55–78%)
	Optic nerve atrophy		22% (9–40%)	**100% (89–100%)**	62% (49–74%)
	Central stalk ^3^		24% (11–42%)	**100% (89–100%)**	63% (50–74%)
Individual pseudoretinoblastoma	PFV/retinal dysplasia	Ciliary body deformations	78% (40–97%)	**95% (86–99%)**	**93% (83–98%)**
		Lens deformations	**90% (56–100%)**	**91% (81–97%)**	**91% (82–97%)**
		Optic nerve atrophy	70% (35–93%)	**100% (94–100%)**	**96% (88–99%)**
		Central stalk ^2^	70% (35–93%)	**98% (91–100%)**	**94% (85–98%)**
	Coats’ disease	Intraretinal macrocysts	38% (19–59%)	**95% (85–99%)**	38% (19–59%)

^1^ Only significant features with sensitivity, specificity or accuracy above 90% (bold) are shown; other analyses are available in Table S2. ^2^ Eye size of (most) affected eye compared with contralateral; ^3^ central stalk from optic disc to lens.

## Supplementary Materials

The following are available online at https://www.mdpi.com/2072-6694/12/12/3592/s1, Figure S1: Adopted MR imaging scoring item list for retinoblastoma, persistent fetal vasculature (PFV) and Coats’ disease, including example images and illustrations, Table S1: Association of individual MR imaging features with diagnosis (retinoblastoma, Coats’ disease, PFV/retinal dysplasia), Table S2: Sensitivity, specificity and accuracy for predicting diagnosis for imaging features significantly associated with diagnosis.
