# Supplementary material for: MR Imaging Features to Differentiate Retinoblastoma from Coats’ Disease and Persistent Fetal Vasculature
Source: Cancers (Basel). 2020 Nov 30;12(12):3592. doi: 10.3390/cancers12123592 (PMC7760210; doi:10.3390/cancers12123592)
Supplement: Supplementary file 1 [file cancers-12-03592-s001.pdf]

Supplementary Materials

Eye	1 Eye size compared to contralateral	Smaller	Same	Larger	
	2 Shallowness of the anterior eye chamber	No		Yes	
	3 Ciliary body deformations	No			
	4 Lens deformations	No		Yes	
	5 Optic nerve atrophy	No		Yes	
Retinal detachment	6 Retinal detachment	No		Yes	
	7 Presence of central stalk between optic disc and lens	No		Yes	
	8 Shape of retinal detachment	T shape	Martini Glass	Sharp V	Wide V
	9 Intensity of subretinal fluid on T2 compared to vitreous	Lower	Equal	Higher	
	10 Intensity of subretinal fluid on T1 compared to vitreous		Equal	Higher	Much higher
	11 Presence of intraretinal cyst-like lesion	No		Yes	
Lesion	12 Presence of solid lesion component (solid appearing lesion or T2 hypointense area)	No		Yes	
	13 Calcification within solid lesion component	No	Possibly	Yes	
	14 Spontaneous T1 hyperintense area within lesion	No		Yes	
	15 Presence of lesion islands (small nodules, T2 dark foci, that are RB seedings or resemble seedings)	No	Yes, in vitreous	Yes, subretinal	
Enhancement	16 Degree of enhancement of solid component (when present)	None	Minimal	Moderate	Strong
	17 Degree of enhancement of anterior eye segment	None	Minimal	Moderate	Strong
	18 Degree of enhancement of uvea (excluding AES) compared to contralateral	Less	Equal	More	
	19 No/minimal contrast enhancement within solid component	No		Yes	
	20 Enhancement mismatch: contrast enhancement outside solid lesion	No		Yes	

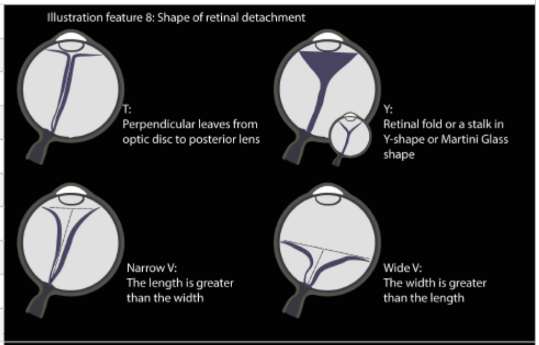

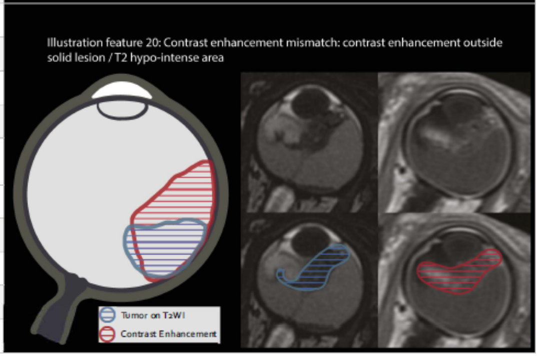

Figure S1. Adopted MR imaging scoring item list for retinoblastoma, persistent fetal vasculature (PFV) and Coats' disease, including example images and illustrations.

Table S1. Association of individual MR imaging features with diagnosis (retinoblastoma, Coats' disease, PFV/retinal dysplasia).

**Feature#**	**MR Imaging feature**	**Fleiss' Kappa for interreader agreement**	**Fisher freeman halton Exact (2-sided) *p* value for association with diagnosis**	**Bonferroni corrected *p* value for association with diagnosis**
1	Eye size compared to contralateral	0.37	<0.001	<0.001
2	Shallowness of the anterior eye chamber	0.71	0.022	0.446
3	Ciliary body deformations	0.39	<0.001	<0.001
4	Lens deformations	0.53	<0.001	<0.001
5	Optic nerve atrophy	0.49	<0.001	<0.001
6	Retinal detachment	0.49	0.476	1.000
7	Central stalk between optic disc and lens	0.43	<0.001	<0.001
8	Shape of retinal detachment	0.40	0.001	0.021
9	Intensity of subretinal fluid on T2 compared to vitreous	0.34	0.021	0.424
10	Intensity of subretinal fluid on T1 compared to vitreous	0.44	0.009	0.180
11	Intraretinal macrocyst	0.55	<0.001	0.005
12	Solid lesion component (solid appearing lesion or T2 hypointense area)	0.26	0.100	1.000
13	Absence of (possible) calcifications	0.35	<0.001	<0.001
14	Spontaneous T1 hyperintense area within lesion	0.21	0.068	1.000
15	Presence of lesion islands (small nodules, T2 hypointense foci that are retinoblastoma seeding or resembling foci)	0.44	0.001	0.018
16	Degree of enhancement of solid component	0.25	0.799	1.000
17	Degree of enhancement of anterior eye segment	0.23	0.042	0.847
18	Degree of enhancement of uvea (excluding AES) compared to contralateral	0.15	0.253	1.000
19	No/minimal contrast enhancement within solid component	0.35	<0.001	0.008
20	Enhancement mismatch: contrast enhancement outside solid lesion	0.15	<0.001	0.001

Table S2. Sensitivity, specificity and accuracy for predicting diagnosis for imaging features significantly associated with diagnosis.

	Sensitivity (95%CI)	Specificity (95%CI)	Accuracy (95%CI)
Smaller eye size predicting pseudoretinoblastoma	71% (53–85%)	91% (76–98%)	81% (69–89%)
Equal eye size predicting retinoblastoma	81% (64–93%)	74% (56–87%)	81% (69–87%)
Larger eye size predicting retinoblastoma	9% (2–25%)	97% (85–100%)	54% (41–66%)
Cilliary deformations predicting pseudoretinoblastoma	30% (65–93%)	100% (89–100%)	66% (53–77%)
Lens deformations predicting pseudoretinoblastoma	38% (22–56%)	97% (84–100%)	68% (55–78%)
Optic nerve atrophy predicting pseudoretinoblastoma	22%(9–40%)	100% (89–100%)	62% (49–74%)
Central stalk predicting pseudoretinoblastoma	24% (11–42%)	100% (89–100%)	63% (50–74%)
T-shape retinal detachement predicting pseudo	31% (15–51%)	77% (59–90%)	55% (42–68%)
Y-shaped retinal detachment predicting pseudoretinoblastoma	28% (13–47%)	100% (89–100%)	65% (52–77%)
Narrow-V-shape retinal detachment predicting retinoblastoma	25% (11–43%)	93% (77–99%)	57% (44–70%)
Wide V-shaped retinal detachment predicting retinoblastoma	55% (36–73%)	66% (46–82%)	60% (47–72%)
Intraretinal macrocysts predicting pseudoretinoblastoma	32% (17–51%)	100% (90–100%)	66% (54–77%)
Absence of (possible) calcifications predicting pseudoretinoblastoma	62% (42–79%)	100% (90–100%)	83% (81–91%)
Vitreous lesion islands/seedings predicting retinoblastoma	30% (16–49%)	96% (78–100%)	58% (44–71%)
Subretinal lesion islands predicting retinoblastoma	64% (45–80%)	76% (58–89%)	70% (57–80%)
No/minimal contrast enhancement within solid component predicting pseudoretinoblastoma	38% (21–57%)	97% (85–100%)	70% (57–81%)
Enhancement mismatch: contrast enhancement outside solid lesion	37% (20–56%)	100% (90–100%)	70% (58–81%)
